# Monitoring Improvement in Infantile Cerebral Palsy Patients Using the 4DBODY System—A Preliminary Study

**DOI:** 10.3390/s20113232

**Published:** 2020-06-06

**Authors:** Krzysztof Krasowicz, Jakub Michoński, Paweł Liberadzki, Robert Sitnik

**Affiliations:** 1 Institute of Micromechanics and Photonics, Faculty of Mechatronics, Warsaw University of Technology, ul. Św. Andrzeja Boboli 8, 02-525 Warsaw, Poland; j.michonski@mchtr.pw.edu.pl (J.M.); p.liberadzki@mchtr.pw.edu.pl (P.L.); r.sitnik@mchtr.pw.edu.pl (R.S.); 2 Patient Care Orthotic Department, Vigo Ortho Poland ul. Oczki 4, 02-007 Warsaw, Poland

**Keywords:** 4D, scanning, movement, rehabilitation, cerebral palsy, treatment, posture, alignment

## Abstract

Monitoring the patient’s condition during rehabilitation is the key to success in this form of treatment. This is especially important in patients with infantile cerebral palsy (ICP). Objective assessment can be achieved through modern optical measurement techniques. The 4DBODY system allows to capture dynamic movement with high accuracy. Eight patients with ICP participated in the study. The group underwent therapy lasting seven days using neurodevelopmental treatment (NDT) and functional training (FT). The patients’ condition was monitored by the 4DBODY system. The measurements were taken three times: before the therapy, after, and then again after one week. We have developed the Trunk Mobility in the Frontal Plane Index (TMFPI) for its assessment. The results were compared with a score obtained using the Gross Motor Function Measure scale (GMFM 88). An improvement of the TMFPI parameter was observed in five patients, inconsistent results in two and deterioration in one. The reference GMFM score was higher in all patients relative to pre-treatment values. We found that surface scanning with the 4DBODY system allows to precisely track body movement in ICP patients. The decrease in the TMFPI parameter reflects the improvement in the dysfunction of body alignment, balance and symmetry of movement on the L and R body side.

## 1. Introduction

Infantile cerebral palsy (ICP) is a disorder which originates in the central nervous system (CNS) in the fetal or perinatal period. According to statistics, its prevalence in Poland is 2 per 1000 live births [1,2].

Infantile cerebral palsy is not a separate disease entity but rather the term identifies a combination of factors that disturb the normal development of a child at a functional and structural level [2]. Damage to the central nervous system causes abnormal distribution of the tonus in the peripheral nervous system (PNS) in the form of muscular hypertonicity, spasticity or hyperactive reflexes mediated by the nervous system [1]. A decrease in muscle strength, impaired movement coordination and balance are also present [3].

These factors may lead to muscle contractures, limited joint mobility and lever-dependent deformations in the osteoarticular system [3]. This condition is reflected in the performance of daily activities in patients and leads to a gradual reduction of their motor activities. It often manifests itself in disorders in the patterns and function of gait [4].

The effectiveness of ICP treatment depends on numerous factors and is associated with interventions at several levels of specialistic action [5]. A significant part of conducting therapy in this group of patients is the correct identification of the root causes of dysfunction and their separation from compensatory mechanisms [1]. In the multidisciplinary model of management, motor therapy plays a major role. However, its effectiveness depends on the proper diagnostic examination of the musculoskeletal system and on objective, systematic monitoring of improvement in treated patients.

The starting point for developing a treatment plan for patients with ICP is to conduct an examination which enables the therapy goals to be determined [5]. Such examination includes structural and functional assessment. In the first case, multiple measures are assessed, e.g., range of motion of joints, length of muscles, power, movement selectivity, muscular tone, and tendon reflexes. Goniometer measurements are used to assess the range of motion of joints [6,7]. It is assumed that the measurement error in this type of tests oscillates within 3 to 5 degrees [8,9]. 

During the functional examination, particular attention is paid to the quality of motor patterns. They are assessed using various types of scales (e.g., GMFCS, MACS, GAS, FMS), as well as functional tests (e.g., the test of stopping before target or the test of maintenance of position) or tasks simulating daily activities (e.g., the UP and GO Test, the Straight Line Walking Test or Passing-By Test) [5,10]. Information from the clinical trial may be supplemented with imaging examinations: X-ray, ultrasound, MRI, CT or three-dimensional gait analysis [3,4]. 

In this model of motor diagnostics, the specialist performing the examination plays the main role. The assessment of the patient’s functional condition depends largely on the experience, knowledge and disposition of a specialist on a given day, which is associated with the risk that the results of the examination may be subjective. This variability strongly influences the starting point for planning the therapeutic procedure and may determine the quality of the planned therapy and the credibility of monitoring improvement during treatment. 

The use of different measurement methods for movement analysis was certainly a step towards the standardization of motor function tests. These include goniometric measurements, video analysis, 3D marker tracking and surface scanning. Some of these methods are supplemented with scales of gait assessment, e.g., by the Edinburgh Visual Gait Scale (EVGS), Visual Gait Assessment Scale (VGAS) and Observational Gait Scale (OGS) [11]. 

Video analysis is the simplest form of such an examination. It is based on recording the patient’s movement with a camera situated in a given plane [12]. As demonstrated by Kawamura, the interpretation of such a recording may differ significantly, depending on the scale used for the assessment and the person by whom the assessment was made [12]. Brown et al. have also confirmed that evaluation of a movement pattern of ICP patients using video analysis largely depends on the experience of the operator and has poor reliability in the knee and hip regardless of the operator’s experience [13]. Moreover, the research by Grunt has shown that video analysis provides repeatable and objective angular assessment of joints, but it can’t be compared with 3D marker tracking due to additional movements in other planes which can’t be measured objectively using video analysis [14]. 

The introduction of the 3D marker tracking method started a new chapter in the field of digital movement analysis. The method uses retro-reflective markers attached to the patient’s body. Their position is determined using multiple cameras and tracked during movement. The markers are mounted at distinctive anatomical points [12]. Marker trajectories enable tracking of the examined object in the form of a skeleton model. This method, often called ‘motion capture’ (MOCAP), enables recording of movement with a frequency ranging from 60 to 420 Hz. The introduction of the MOCAP method served to develop a more precise analysis of movement and capture even subtle changes in movement patterns [12]. Currently, MOCAP is an examination used clinically to assess the patient’s condition in the overall treatment process, which should result in improved gait function [12].

Interpretation of the obtained examination results depends on the objectives of the therapy. Meyns et al. studied the influence of upper extremities on the movements of lower extremities during gait using MOCAP [15]. Dixon et al. used MOCAP to examine children with ICP suffering from disturbances of gait direction and central stabilization [16]. Examinations with the use of MOCAP are certainly more accurate and demonstrate higher repeatability than video analysis [17]. 

The use of skin markers has been identified as one of the main disadvantages of this method [18]. Attaching markers to the skin is a time-consuming process and unnecessarily extends the duration of the examination. In addition, skin markers change their position on the skin during movement, which increases the inaccuracy in the mapping of the skeletal system [19].

The latest chapter in the recording of human movement began in 2010, when Microsoft released the Kinect sensor [20]. The motion sensor consisted mainly of an RGB camera and a depth sensor for recording motion without any additional hand-held controllers or body-mounted artifacts. Kinect enabled recording at a frequency of 30 Hz. The depth detector allowed the reproduction of scanned objects in a three-dimensional coordinate system. The reconstruction method used an infrared camera and a pattern-emitting projector. In 2013, Microsoft released an improved second version of the sensor. The device was equipped with a time-of-flight depth sensor and provided better quality data, both in raw depth measurements and skeleton approximations [21]. With low costs, easy handling and a large supply, the system quickly became popular in applications focused on movement evaluation. 

Many researchers have investigated the possibility of using Kinect in the study movement patterns. Rocha AP et al. studied gait assessment using the second version of the Kinect sensor. In this study, the system was recognizing whether an object was moving or not in relation to different measuring tasks (walking toward and away from sensor) and distances (7 and 5 m). Moreover Rocha reported that the algorithm used for gait analysis achieved errors for heel strike instant and stride duration estimation of 15 ± 25 ms and 1 ± 29 ms (walking towards the sensor), and 12 ± 23 ms and 2 ± 24 ms (walking away from the sensor). The obtained data was similar to the results from clinically proven MOCAP systems and show that RGB-D cameras can be used for gait analysis [22].

Knippenberg et al. investigated the effectiveness of a markerless surface scanning system in the rehabilitation of patients with pathological conditions of the nervous system [23]. Jin Tang et al. studied the effectiveness of the use of a markerless 2.5D measurement system for registration and description of gait cycles [24].

However, the possibility of using currently sold scanning systems for objective assessments under clinical settings is limited. [20,25,26] Examination using 3D surface measurement systems is a relatively quick procedure and does not require any markers to be attached to the patient’s body [27]. The quality of generated data seems to be the main problem, especially in the low-end scanning systems. For this reason, assessing the location of the distinctive anatomical structures is difficult, and tracking their movement is even more difficult [27]. Accurate 3D scanning systems only allow the recording of static, often small objects, which limits their use [27].

The dynamic 3D scanning method has set a new direction in motion analysis. The US-based company 3DMD, which developed a system for recording movements of anatomical structures with a frequency of up to 120 Hz, is a leader in this field [28]. Their system uses a method of surface scanning with LED lighting for capturing movement without the need for markers. The potential of this technology is very wide. Among other things, it found application in medicine, e.g., in orthodontics for examining facial asymmetry and skull deformation in children, and in the field of esthetic supplementation of facial defects in patients with neoplasms [29]. This technology also allows you to scan the entire person during free movement without the need to prepare scanned surfaces or attach markers. 

A study published by Lenar confirms the effectiveness of this method [25]. The solution proposed by the authors is based on 4D scanning technology using a structured light illumination method. The system records movement with a frequency of up to 60 Hz, spatial resolution of 2 mm and inaccuracy of 2 mm. The system was the first version of the current 4DBODY system and was used for assessment of kinematics of lower extremities during movement. The authors showed good correlation between selected gait parameters registered using the developed method and the marker-based measurement [25]. Gait parameters were calculated based on the movement of virtual markers determined from 4D scanning data. This work proved the potential of using surface data and at least their equivalence with data from a state-of-the-art marker system. In another study, based on the system described by Lenar, Liberadzki presented the possibility of using the 4D technique to scan people during movement. The 4DBODY scanner with a recording frequency of 120 Hz, a spatial resolution of 1mm and an inaccuracy of 0.5 mm was developed for this purpose [30]. The output from the scanner is a sequence of point clouds representing the body surface in motion. Such a high measurement resolution enables precise reconstruction of body movement in patients with various types of motor dysfunctions. The described system is used in the current study as a measurement tool. 

In our opinion, the 4DBODY system might be an excellent diagnostic tool that will be a valuable addition to examinations, at both the functional and structural level. This will help objectivize the assessment of the patient’s condition and achieve high repeatability of the tests performed. Due to high accuracy, non-invasiveness, and ability to record high dynamics of movement, this system can be widely used for practical assessments of rehabilitation effects. This, in turn, would provide an opportunity to objectively assess the patient’s condition and make adjustments to the motor function therapy plan. 

We propose a novel use of the 4DBODY system as a tool for assessing movement parameters in patients with ICP, which may lead to the development of an objective method of movement analysis based on surface measurements. To prove its potential, we also propose the TMFPI (Trunk Mobility in the Frontal Plane Index), a novel algorithm for evaluating movement sequences of ICP patients captured using the dynamic 3D method. We perform preliminary validation of the algorithm by comparing the results with the well-known GMFM-88 scale.

## 2. Materials and Methods

Eight patients with diagnosed infantile cerebral palsy (ICP) in the form of spastic hemiplegia and diplegia participated in the study. The parents’ written consent was obtained. Four patients were male, four female, and the average age was 9 ± 2 years. Seven subjects were rated as level II according to the GMFCS classification (Gross Motor Function Classification System) and one subject as level III. 

The children selected for this study met the following inclusion criteria: age 5 to 18 years old, diagnosis of ICP (diplegia or hemiplegia), walking with or without assistance of aids (GMFCS I-III), attending rehabilitation sessions regularly (at least 2 times per week), and participating in functional skiing training sessions (at least 2 sessions per week). Exclusion criteria were: GMFCS IV-V, symptoms of dystonia or ataxia, epilepsy.

Seven patients wore bilateral AFO orthoses for a minimum of 6 h a day and one patient did not use any lower limb orthopaedic equipment. Seven children did not use any additional orthopaedic equipment for the remainder of the period. One child used a mobile walking aid for longer distances, although unaided walking was possible. The characteristics of the group are presented in Table 1.

Incorrect control of posture within the trunk during standing and walking, and during other activities related to the alternating work of the extremities, was found in all subjects, based on observations and clinical examinations. These motor deficits were assessed, among others, on the basis of the Duchenne sign and the Trendelenburg sign, visible during the examination. All patients were included in the motor function rehabilitation program in accordance with the neuromuscular development therapy (NDT) approach at the rehabilitation center [31]. In addition, all patients have undergone a rehabilitation cycle based on parts of functional training (FT) including downhill skiing technique elements [32].

The training was conducted on a ski slope by physiotherapists who were also downhill skiing instructors. Patient rehabilitation was conducted for 7 days. Every day, patients had 2 h of NDT and 2 h of skiing technique training. The therapy focused on improving the central stabilization within the trunk in the frontal plane by strengthening the abdominal muscles, extensors of the hip joints, with particular emphasis on the middle gluteal muscles. Attention was also paid to stretching of the erector muscles of the spine, flexors of the hip and knee. It was assumed that the balance between weakened muscles and muscles shortened due to spasticity may significantly influence the quality of movement. 

## 3. Methods

### 3.1. Study Design

The experiment followed a repeated measures design, three conditions were examined:

- before the rehabilitation cycle,
- at the end of the rehabilitation cycle,
- seven working days after the end of the rehabilitation cycle.

The choice of these three moments made it possible to compare the condition of patients before and after the rehabilitation session and to assess whether the therapeutic effect persisted after its discontinuation.

### 3.2. Measurement Procedure

The study was conducted using the 4DBODY system [30]. The system was developed by our group and could provide measurements of the 3D geometry of body surface during movement, with independent acquisition and processing of each recorded frame resulting in a series of 3D point clouds. The system uses a single-frame structured light illumination method which allows the registration of the shape of body surface with a frequency of up to 120 Hz [30]. It consists of eight measurement heads, each with an LED projector and a CCD camera, spectrally separated within the visible spectrum. The heads were arranged around a measurement stage (1.5 m × 1.5 m × 2 m) on which the patients performed requested movements. The setup proved to be effective, although other approaches are also possible [33]. The heads were connected to a synchronization module, which allowed for precise measurements with all the individual heads simultaneously. The result of the measurement was a cloud of points (XYZ) describing the body surface with a resolution of 1 point per square millimeter. 

**Figure 1:**
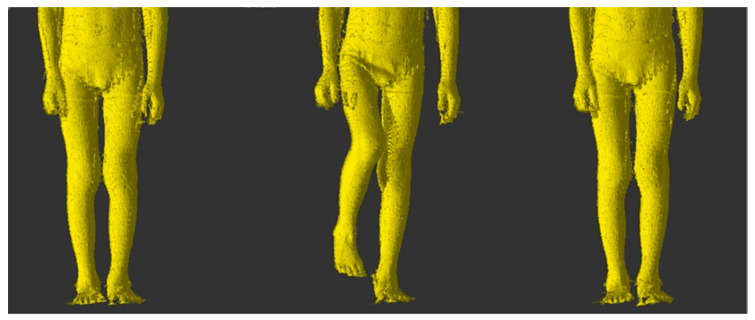
Hip flexion in the frontal plane-transition from initial position to maximum flexion and return.

The 4DBODY system was used to scan movement sequences, in which trunk stabilization during the change from standing on two legs to standing on one leg (Figure 1) was assessed. Three measurement sessions were carried out according to the experiment plan. Each consisted in a measurement of the whole body. Patients did not use any orthopedic equipment or other aids that improve trunk stabilization during the measurement.

**Figure 2:**
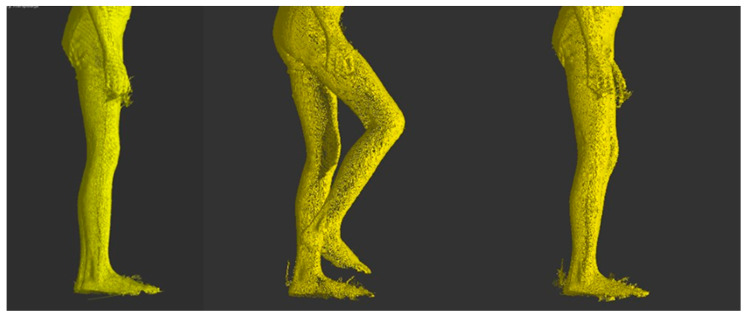
Hip flexion in the sagittal plane-transition from initial position to maximum flexion and return.

For the measurements the subject was asked to take the initial position, perform a hip joint flexion in the maximum possible range, and return to the initial position (Figure 2). First, the 4DBODY system operator began recording the measurement sequence. Immediately afterwards, the patient received a voice signal to begin the movement. After the movement finished, the operator stopped recording.

### 3.3. Data Analysis

In order to investigate changes in patient’s movement patterns across the measurement sessions, trunk shift in the frontal plane during maximum hip joint flexion was assessed. The choice of this modality was based on the authors’ observations of the body kinematics of ICP patients. We propose the Trunk Mobility in the Frontal Plane Index (TMFPI) to describe these changes. 

The FRAMES software package, developed at the Institute of Micromechanics and Photonics, Faculty of Mechatronics of the Warsaw University of Technology [34], was used to extract the relevant data from 4D measurement sequences.

As a preprocessing step, the sequences were aligned according to the anatomical planes. This procedure is not part of the proposed algorithm, however, the correct determination of anatomical planes is crucial for obtaining the correct results. 

First, the vertical position was determined using a calibrated plumb line. Alignment of the frontal plane was achieved by rotating the entire sequence in space around the vertical line. The goal was to place the most salient points of the buttocks from the first frame of the sequence in the frontal plane. The first frame was chosen because of the maximum similarity to the standing position. The salient points were semi-automatically determined based on buttock areas that were manually marked by the operator. A best fit plane was determined from the selected most salient areas and its normal vector was used to calculate the additional rotation.

Determination of the TMFPI required manual selection of three frames from the recorded sequence, corresponding to three phases of movement:

- beginning of movement–i.e., the initial position when the patient stands still with the arms along the trunk and waits for the command to start the movement,
- middle phase of movement–the moment of maximum hip flexion; the goal was to capture the moment when the limb was in its highest position,
- end position–means returning to the initial position, i.e., standing on two legs.

In the next steps, only the chosen frames were processed. Noise filtering was performed using a DBSCAN-type clustering algorithm implemented in the FRAMES environment [35]. The algorithm was used to remove small groups of points. The set minimum distance between clusters was 2 mm. Groups smaller than 2000 points have been removed. 

**Figure 3:**
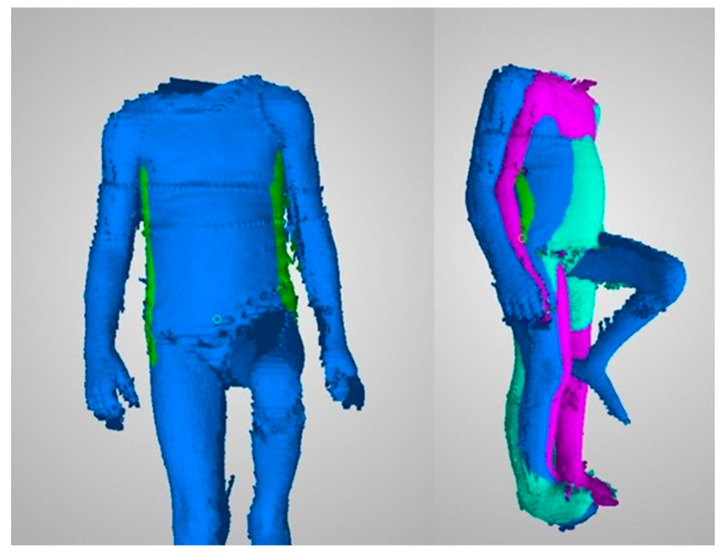
Left hand side: a single frame in the frontal plane with marked trunk outline. Right hand side: three frames with individual movement phases.

Then the operator roughly marked the left and right body outline in the frontal plane. The segments were marked approximately from the greater trochanter of the femoral bone to the level of the axillary fossa. Trunk fragments were marked using a brush-type tool. Finally, all the points marked have been exported to external files in the form of an XYZ cloud oriented according to the anatomical planes (Figure 3). The exported sets contained only points on the trunk outline.

**Figure 4:**
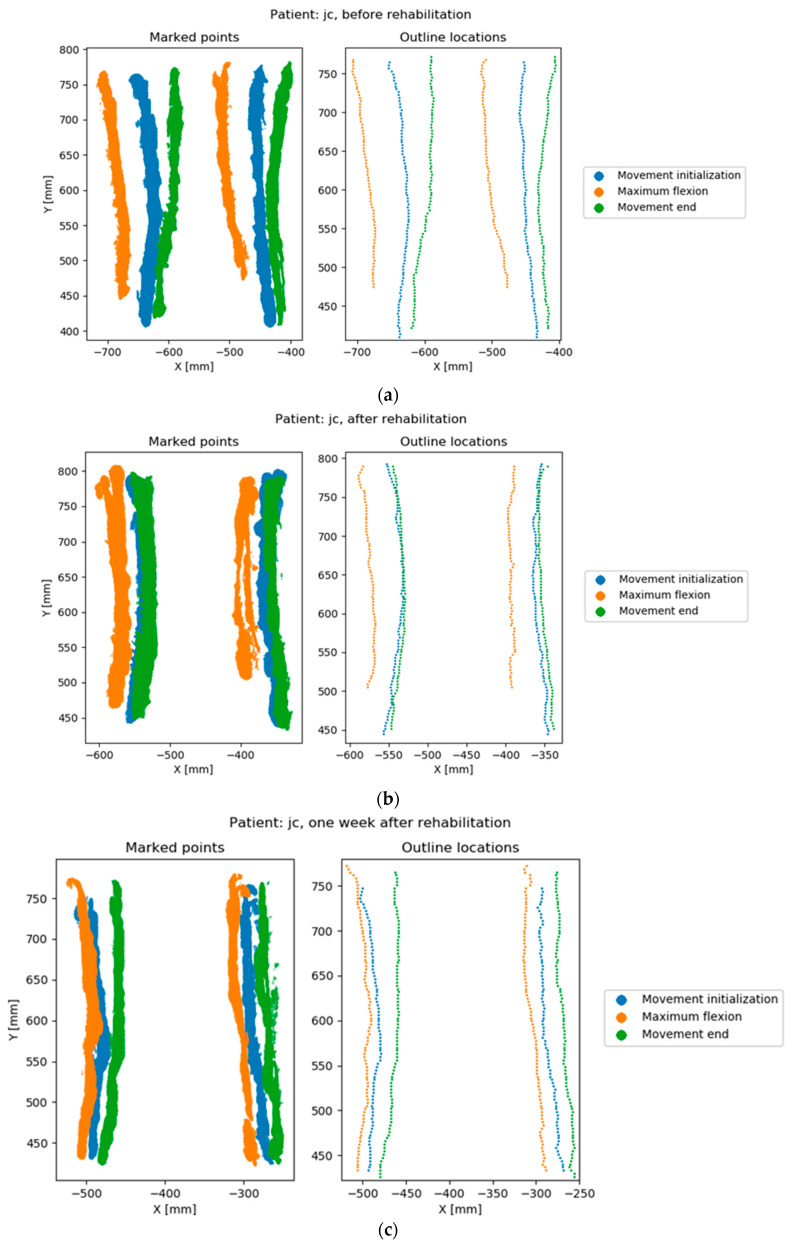
Points marked by the operator and calculated outline locations. Rows correspond to the measurement sessions of the same patient; (**a**) before rehabilitation, (**b**) after rehabilitation, (**c**) one week after rehabilitation.

The last part of the algorithm has been implemented in Python. Sets of three frames corresponding to the three phases of movement were analyzed using the following procedure (Figure 4).

**Figure 5:**
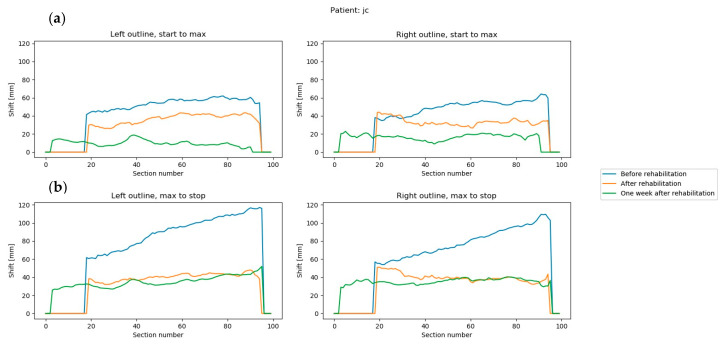
Trunk movement in all three measurement sequences of a single patient (**a**) difference between the first and middle frames, left and right outline; (**b**) difference between the middle and end frames, left and right outline. Section number is the number of the horizontal slice, ranging from 0 to 100.

(1) The total horizontal coordinate range in the sequence was determined.
(2) Each frame was preprocessed separately using the operations:
  (a) Rough determination of a vertical line separating the left outline from the right outline. The line was determined with a simple recursive subdivision scheme in the horizontal axis to avoid noise.
  (b) Division into 100 horizontal sections of the same height. The horizontal range covered by the sections was the same in all three frames and corresponded to the horizontal range calculated in Step 1. This meant that some top or bottom sections in some of the frames could be empty. Each section corresponds to a percentile of the analyzed range, which facilitates interpretation. The approximate section height in the analyzed examples was around 1 cm.
  (c) Division within a section into left and right outline. The division was made by finding a horizontal midline using the iterative method described in Step 2a.
  (d) Removal of noise sections. Noise sections were defined as:
    - sections without a left or right outline, identified as having a width less than 30% of the horizontal coordinate range,
    - sections with insufficient number of points, identified as having less than 10 points in the left or right outline
    - noise sections can be identified by other criteria, depending on the specific nature of the measurement data.
  (e) Determination of two points in each section that indicate the position of the left and right outline in this section. These points were determined as the arithmetic means of the points representing the left and right outline.
(3) The offset between the points representing the left and right trunk outline in each section was determined between pairs of frames. Two pairs were used: the first and middle frame and the middle and end frame. These differences represent the trunk movement in the frontal plane between the initial and highest position of the limb, and between the highest position and movement end. High values of left or right trunk outline shift within a section represent a large trunk shift at this location during movement (Figure 5).
(4) Final value of the TMFPI was determined for the whole sequence by calculating a quadratic mean of all offsets obtained in Step 3. The index can also calculated for subparts of the movement, describing the amount of trunk movement:
  - between the initial and mid part of the movement–TMFPI_12,_
  - between the mid and final part of the movement–TMFPI_23,_
  - only on the left side–TMFPI_L_,
  - only on the right side–TMFPI_R_.

The TMFPI value is directly related to the trunk movement in the frontal plane within the range marked by the operator. The index is expressed in millimeters and is always positive, a higher value corresponds to a larger change within the trunk during the analyzed part of the movement. Therefore, the TMFPI provides information only about the amount of movement, without its direction. 

### 3.4. Ground Truth

**Figure 6:**
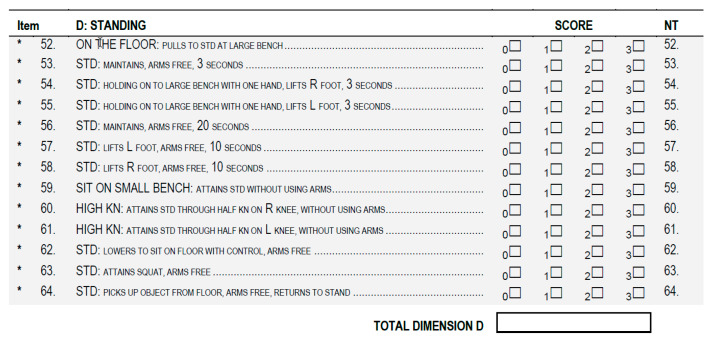
The GMFM-88 scale dimension for standing activities assessment. (GMFM-88, 2013 Dianne Russell and Peter Rosenbaum, McMaster University, Hamilton, ON, Canada).

**Figure 7:**
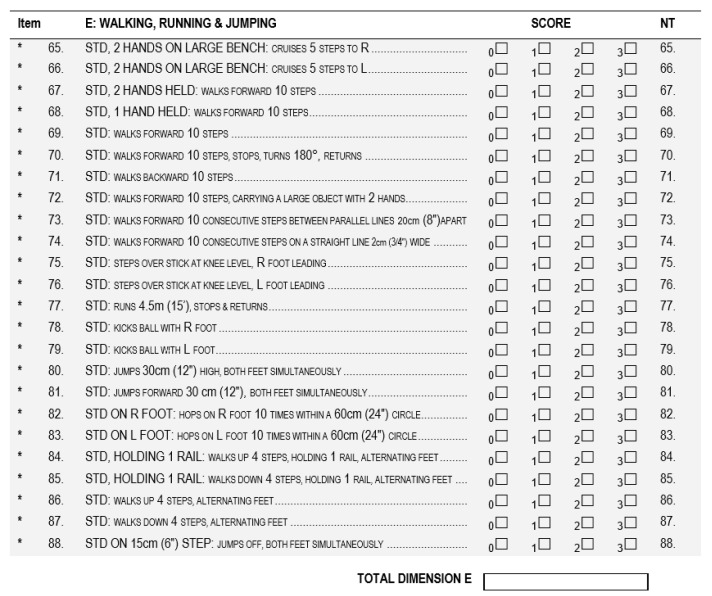
The GMFM-88 scale dimension for walking activities assessment. (GMFM-88, 2013 Dianne Russell and Peter Rosenbaum, McMaster University, Hamilton, ON, Canada).

The obtained TMFPI values were compared with the assessment according to the GMFM-88 (Gross Motor Function Measure 88) scale [36], performed before and after the rehabilitation cycle. During the assessment, the subjects were instructed to perform the same movement as for the 4DBODY system. Particular attention was paid to the trunk stabilization during standing and walking (dimension D and E) (Figure 6 and Figure 7). 

During the examinations, a physiotherapist assessed each motor task according to a 4-grade rating scale and assigned the following ratings to a given movement:

- 0 when the movement was not performed.
- 1 when the movement was initiated by the subject.
- 2 when the movement was performed, but not to the full extent.
- 3 when the movement was performed to the full extent.

If an activity was not evaluated in a given patient, the assessment of this dimension in the score sheet was not entered. The quality of the motor task corresponds to the sum of points obtained from the assessment. On this basis, the final result was calculated according to the following formulas:

$$\mathrm{Dimension}\;D=\frac{\mathrm{sum}\;\mathrm{of}\;\mathrm{partial}\;\mathrm{scores}\;\mathrm{from}\;\mathrm{movement}\;\mathrm{assessment}}{39}\times 100\%=\mathrm{final}\;\mathrm{result}\;\%$$

$$\mathrm{Dimension}\;E=\frac{\mathrm{sum}\;\mathrm{of}\;\mathrm{partial}\;\mathrm{scores}\;\mathrm{from}\;\mathrm{movement}\;\mathrm{assessment}}{72}\times 100\%=\mathrm{final}\;\mathrm{result}\;\%$$

## 4. Results

**Figure 8:**
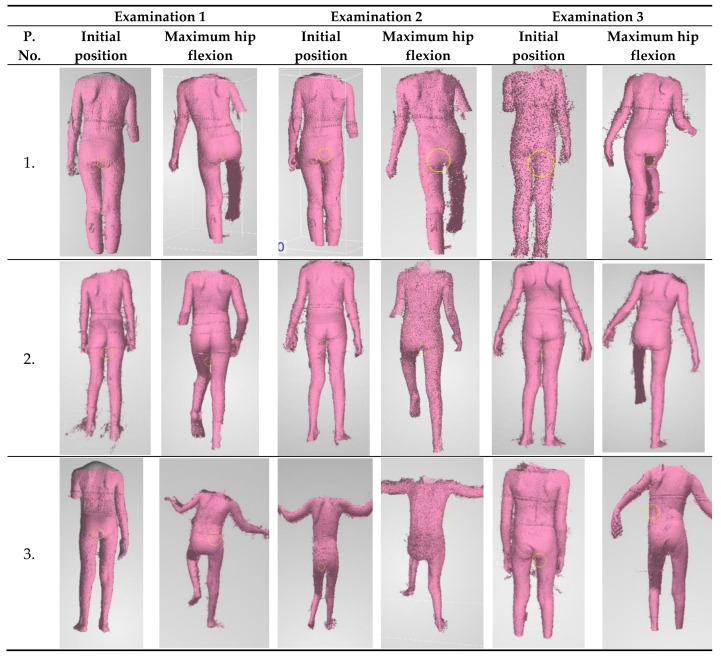

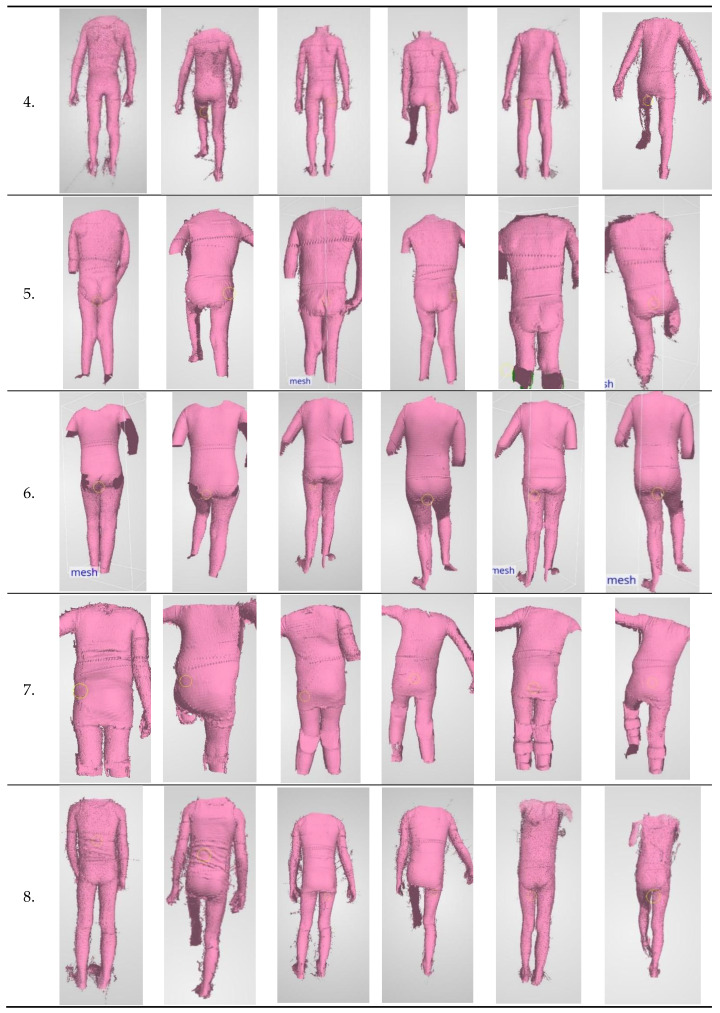
Exemplary scans of patients from individual measurements.

ICP patients undergoing rehabilitation were the studied test group. An attempt was made to assess the impact of motor function rehabilitation on changes in trunk stabilization in the frontal plane. Exemplary scans of the movement sequence performed during three measurements sessions are presented in Figure 8. 

**Figure 9:**
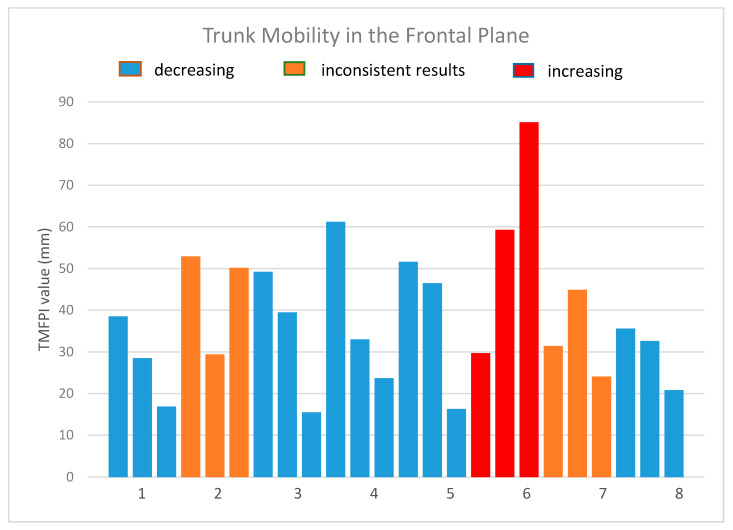
The values of the trunk mobility in the frontal plane index.

A total of 24 measurement sequences were collected. Sequence duration was 1.3 ± 0.5 s. Values of the TMFPI obtained for the sequences are presented in Table 2 and Figure 9.

Three tendencies could be identified in the obtained results:

- in five patients (patients 1, 3, 4, 5, 8) the 4DBODY system recorded a decreasing TMFPI value in subsequent measurement sessions,
- variable change of the TMFPI in two subjects (patient 2, 7); in one case the middle session has the lowest value, while in the other it has the highest value; however, in both cases the TMFPI value decreased between the first and last measurement session,
- measurements in one subject showed an increasing TMFPI value.

**Figure 10:**
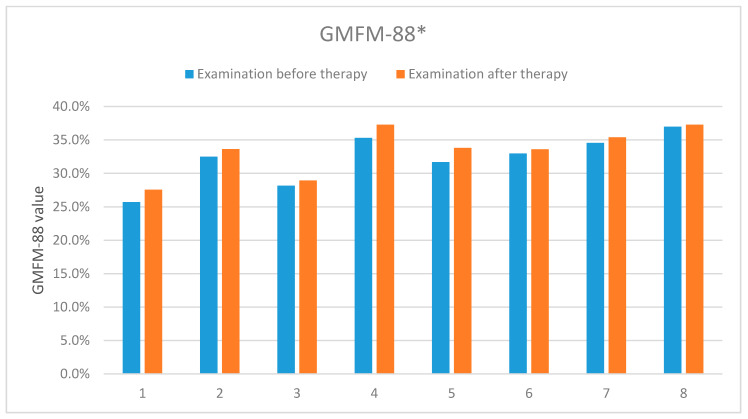
The results of the according to the GMFM-88 scale carried out before and after the rehabilitation cycle. *-concerns dimensions D and E of the measurement sheet (GMFM-88, 2013 Dianne Russell and Peter Rosenbaum, McMaster University, Hamilton, ON, Canada).

The results presented in Table 3 and Figure 10 describe the first and second evaluations according to the GMFM-88 scale, corresponding to the first and second measurements with the 4DBODY system. The final GMFM-88 score is calculated as the mean of its components (A − E). In our case, components A, B and C were equal zero, which resulted in the final relation of (D + E)/5. 

**Figure 11:**
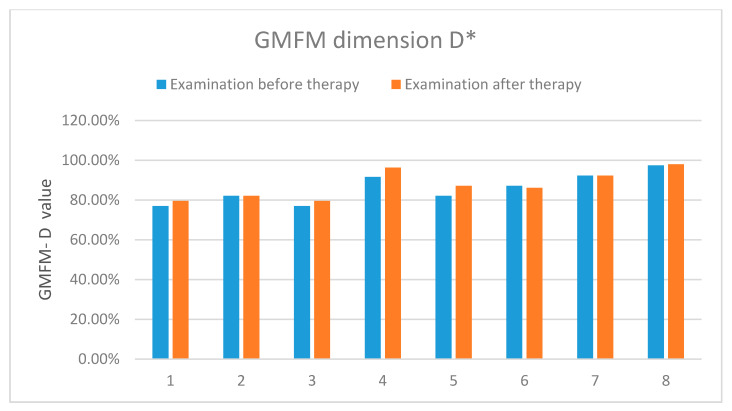
The results of the assessment according to the GMFM-88 scale-dimension D. *-concerns the dimension D of the measurement sheet (GMFM-88, 2013 Dianne Russell and Peter Rosenbaum, McMaster University, Hamilton, ON, Canada).

The results showed an improvement in the functional status in all the examined patients. Table 4 and Figure 11 present detailed results of the assessment using the GMFM-88 scale only for the dimension D.

**Figure 12:**
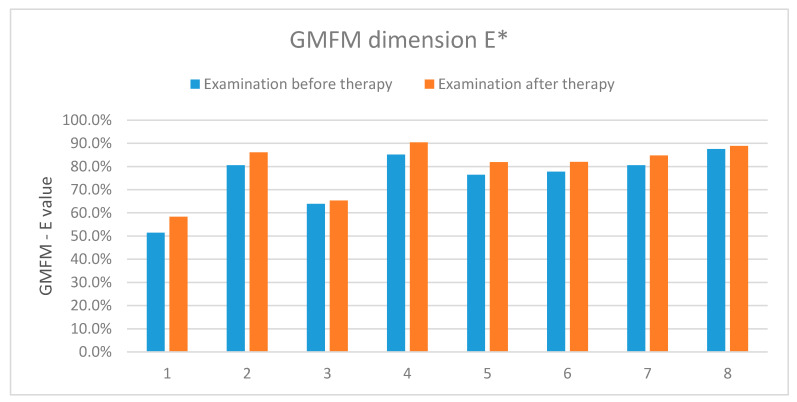
The results of assessment according to the GMFM-88 scale-dimension E. *-concerns the dimension E of the measurement sheet (GMFM-88, 2013 Dianne Russell and Peter Rosenbaum, McMaster University, Hamilton, ON, Canada).

The results showed an improvement in the functional status in the five examined patients, two presented no improvement and one-deterioration. Table 5 and Figure 12 present detailed results of the assessment using the GMFM-88 scale only for the dimension E.

The results showed an improvement in the functional status in all the examined patients.

## 5. Discussion

The aim of this study was to investigate the usefulness of the 4DBODY system for monitoring the condition of patients with pathological conditions of the nervous system undergoing motor function rehabilitation. 

Infantile cerebral palsy is a combination of factors associated with injuries of the central nervous system that cause disorders of the musculoskeletal system. Such dysfunctions may result in permanent changes in the osteoarticular system. However, properly conducted diagnostic examinations of the motor function, including clinical examinations, allow to carry out rehabilitation that can effectively counteract these changes. 

A common assumption is that objective assessment of improvement during therapy has a large impact on its effectiveness. Whether dynamic 3D scanning technology could be useful for objectively recording changes in patients, especially those suffering from ICP, remains an open question. To successfully capture this information, the measurement sequence must be acquired with sufficient precision and the data processed in such a way, that interpretable numeric values can be presented. 

Measurements of movement sequences of eight children with ICP using the 4DBODY system, which implements this technology, were used to develop a new index, the TMFPI. The index was used to assess changes in these children on a per-case basis. The obtained results were compared with one of the commonly used and clinically approved functional assessment scales–the GMFM-88. 

During the examination the subjects were asked to change their position from support on two legs to support on one leg. Under such conditions, the pelvis with the supporting leg became a type-I lever. Keeping the pelvis horizontally in the frontal plane required the activity of the middle gluteal muscle on the side of the supporting leg. Insufficient power of this muscle caused a disturbance in the lever system. The pelvis on the opposite side was dropping in relation to the supporting leg and the patients compensated the pelvis position through trunk inclination to the opposite side in the sagittal plane.

This mechanism occurred when the subjects performed a flexion of the hip joint. The change to standing on one leg was introduced to reduce trunk stability by reducing the support surface. It was assumed that such a movement pattern would require a higher degree of central stabilization in the examined patients. The movement from the initial to the middle position acted against the gravitational force and therefore required more effort.

During the second part of the movement, when the leg returned from the middle position to the end position, the gravitational force acted in the direction of movement, making the return easier and faster. This had a significant impact on the speed of the entire movement and on the trunk stability in this examination.

The sequence was a complex movement for the patients. Performing this task required shifting body weight to the supporting leg in order to reduce the load on the leg performing the hip flexion. The supporting leg had to ensure stabilization of the whole body under these conditions, in particular the trunk. The correctness of the movement depended on the stability of this segment.

It is worth noting that the very fact of conducting the examination affected some patients. In several cases, the subject was already preparing for the examination, slightly reducing the load on the leg to be flexed, waiting for the signal to initiate movement.

Another point worth highlighting is the observed impact of the time of day in which the measurement was carried out. If the measurement session took place in the afternoon, after school lessons or rehabilitation therapy, the patient’s motor abilities were significantly reduced compared to the same measurements carried out in the morning. The patients had more difficulty in performing the investigated movement. During the examination, the concentration dropped significantly and the trunk stabilization deteriorated.

This is important due to the fact that patients have been examined without their daily orthotic equipment. Wearing orthoses resulted in better stabilization in the distal segments of the biomechanical chain, which led to better central stabilization. Therefore, during examinations without orthoses, patients had to control the position of the lower limbs much more than if they wore orthoses to which they were accustomed. 

Examination using the 4DBODY system showed deterioration in all the measurement sessions in the oldest patient in the study group. It should be noted that for a long time he did not use any lower limb orthoses, which led to permanent changes in the osteoarticular system. Uncorrectable deformations of the feet, and flexions of the knee and hip joints made changing to standing on one leg very difficult. In this case, the correct hip joint flexion was seriously hindered by the trunk, a large biomechanical segment whose control was difficult while standing on two legs. This movement caused a triple flexion position of the lower limbs and forward inclination of the trunk. Such conditions caused an imbalance, directly affecting the hip flexion range. Performing a hip flexion resulted in a compensatory mechanism in the form of lateral movements of the trunk.

Examination results obtained using the 4DBODY system were compared with the assessment made according to the GMFM-88 scale. This scale is a generally approved tool for the assessment of changes in the gross motor function in patients with ICP. It was chosen as ground truth for verification of the results from the 4DBODY system. For the purpose of assessing the trunk stabilization during hip flexion, we focused on the dimension D of GMFM, which relates in particular to standing activities. One of the motor tasks from dimension D according to this scale, consisting in lifting the foot from the ground during unaided standing, was in line with the examined by 4DBODY system hip flexion movement.

It was characteristic of the examined group that they achieved maximum results in this task, provided they lifted the foot while holding on to a bench or chair. The trunk stabilization in these conditions improved significantly, which directly translated into better control of performed lower limb movements. This motor task was much more difficult without hand stabilization because it required active stabilization within the trunk. 

Five patients with better trunk stabilization according to the GMFM scale–dimension D had a similar tendency in the TMFPI values obtained from the 4DBODY system. The examination carried out after the rehabilitation cycle showed improvement by one point according to the assessment scale used in the GMFM score sheet.

Two patients with improved TMFPI values only between the 1st and 3rd measurement session had also no improvement according to the GMFM dimension D assessment. Based on the observation of movement during examinations, asymmetrical lower limb work was noticed in these patients, manifesting in a difference in the hip joint flexion angle with respect to the opposite limb. At the same time, incomplete, asymmetrical extension in the knee joint was observed in the supporting leg. We consider these deficits in the extension of the knee joints as a significant factor in the quality of hip joint flexion and stabilization of the torso. After rehabilitation, the asymmetry did not change, so the result of the second measurement was similar. This confirmed earlier observations regarding the adverse effect of this factor on central stabilization. The pelvis was positioned in anteversion and the patients, striving to create the best stabilization within the trunk, additionally deepened the lordosis of the lumbar segment of the spine.

In the patient in which the 4DBODY system showed a gradual increase in the TMFPI values, the symptoms returned also according to the GMFM dimension D scale in relation to the assessed movement. The first measurement showed a clear asymmetry between the left and right sides of the body. The left leg movement was only initiated and the right leg movement was not completed. The second evaluation after the rehabilitation cycle showed deterioration of the results on the left and right leg, reflecting only to a small extent the initiation of the assessed movement.

Sequences captured using the 4DBODY system were analyzed according to the proposed Trunk Mobility in the Frontal Plane Index. The authors used their best knowledge to select the appropriate factors in the developed movement analysis to make it as objective as possible and reflect significant changes for the examined condition. However, calculation of the TMFPI from the movement sequences is not fully automatic and requires the involvement of an operator with medical background. This can be interpreted twofold. On the one hand, full automation removes dependence on a qualified operator and generally allows to achieve a higher level of objectivity. On the other hand, the current approach separates the problem of objective assessment from the requirement of correct anatomical interpretation. In this case, it is the responsibility of the operator to correctly align the frontal plane and choose the appropriate segment of the trunk. Provided that this information is correct, the algorithm allows for objective evaluation of the input data. 

Nevertheless, special attention should be paid to the sources of uncertainty of the developed algorithm. Most of them are related to the manual marking of areas from which the proposed index is determined. Moreover, the results will be influenced by:

- the accuracy of the plumb line calibration,
- position of the frontal plane,
- the number of sections used in the analysis,
- parameters used for filtering out noise sections.

Also, some variability will be associated with measurement noise from the 4DBODY system. The extent to which these factors affect the final result should be considered in the future.

## 6. Conclusions

This paper presents preliminary results of examining the possibility of using precise dynamic 3D scanning, in particular with the 4DBODY system, for assessment of the rehabilitation process of patients with pathological conditions of the nervous system. The choice of the group of patients for the study was not accidental. Positive evaluation of the usefulness of the measurement system in patients with deficits of the nervous system, in whom regular and objective monitoring of the rehabilitation process is particularly important, may be the key to ensuring better quality of therapy in this group. 

Verification of assumptions and achievement of therapeutic goals is a necessary basis for effective treatment of ICP patients. Early detection of unwanted compensatory mechanisms can effectively limit the extent of deformations of the musculoskeletal system.

The conducted assessments indicate that the use of dynamic 3D surface measurements is a promising direction of research and can provide valuable information on patient movement patterns. The proposed method allows for semi-automatic determination of the Trunk Mobility in the Frontal Plane Index (TMFPI) and enables assessment of changes in trunk stabilization. The developed index provides results agreeable with the clinical indicator GMFM-88 and with clinical observations of a physiotherapist. According to our evaluation, motion analysis based on measurements with the 4DBODY system would allow for detailed monitoring of changes during complex processes of treatment of ICP patients and for such a selection of methods that will optimize its effect.

Development of this type of analysis methods, their automation and objectivization, as well as further in-depth verification including repeatability and uncertainty assessment will be the goals of our studies in the future. We would also like to study a larger group and increase the number of measurement sessions. 

Our future goals include evaluating the effectiveness of lower limb orthoses in ICP patients, which may enable their quick and precise adaptation to changing individual biomechanical conditions. A thorough analysis of movement patterns for lower limb orthoses would make it easier to decide if this method of treatment is necessary for some patients. 

Also, another direction of research would be how to extend the verification of the 4DBODY system with respect to a clinically approved MOCAP system. Large volumes of data provided by the 4DBODY system during movement may be an alternative to marker systems currently used for gait evaluation. The precise surface representation obtained from the system can also be used in the design and creation of individual orthopaedic equipment through 3D printing. 

The dynamic 3D surface scanning technology certainly opens a new chapter in the modern history of physiotherapy, enabling effective and targeted therapeutic management that can be planned and verified at every stage of treatment. In the future, after reaching practical maturity, the method described in the work can be used in more complex configurations of the Healthcare Monitoring System [37].

## Figures and Tables

**Table 1:** Characteristics of the group of subjects in the experiment.

Patient Number	Sex	Age	Diagnosis	Type	Use of Lower Extremity Orthosis *	GMFCS
1	f	8	ICP	Hemiplegia	Yes	2
2	m	9	ICP	Diplegia	Yes	2
3	m	9	ICP	Hemiplegia	Yes	2
4	m	11	ICP	Diplegia	Yes	2
5	m	5	ICP	Diplegia	Yes	2
6	f	16	ICP	Diplegia	No	3
7	f	8	ICP	Diplegia	Yes	2
8	f	9	ICP	Hemiplegia	Yes	2

* For a minimum of 6 h a day.

**Table 2:** Values of the Trunk Mobility in the Frontal Plane Index (millimeters).

TMFPI	Patient 1	Patient 2	Patient 3	Patient 4	Patient 5	Patient 6	Patient 7	Patient 8
Meas. 1	38.5	52.9	49.2	61.2	51.6	29.7	31.4	35.6
Meas. 2	28.5	29.4	39.5	33.0	46.5	59.3	44.9	32.6
Meas. 3	16.9	50.2	15.5	23.7	16.3	85.1	24.1	20.8

**Table 3:** The results of the assessment according to the GMFM-88 scale (dimensions D and E) carried out before and after the rehabilitation cycle.

GMFM-88 (D and E)	Patient 1	Patient 2	Patient 3	Patient 4	Patient 5	Patient 6	Patient 7	Patient 8
Exam. 1	25.7%	32.5%	28.2%	35.3%	31.7%	33.0%	34.6%	37%
Exam. 2	27.6%	33.6%	29.0%	37.3%	33.8%	33.6%	35.4%	37.3%

**Table 4:** The results of the assessment according to the GMFM-88 scale (dimension D) carried out before and after the rehabilitation cycle.

GMFM-88 (Dimension D)	Patient 1	Patient 2	Patient 3	Patient 4	Patient 5	Patient 6	Patient 7	Patient 8
Exam. 1	76.9%	82.1%	76.9%	91.6%	82.1%	87.2%	92.3%	97.4%
Exam. 2	79.5%	82.1%	79.5%	96.3%	87.2%	86.2%	92.3%	97.9%

**Table 5:** The results of the assessment according to the GMFM-88 scale (dimension E) carried out before and after the rehabilitation cycle.

GMFM-88 (Dimension E)	Patient 1	Patient 2	Patient 3	Patient 4	Patient 5	Patient 6	Patient 7	Patient 8
Exam. 1	51.4%	80.6%	63.9%	85.2%	76.4%	77.8%	80.6%	87.5%
Exam. 2	58.3%	86.1%	65.3%	90.4%	81.9%	81.9%	84.7%	88.9%
